# Qualitative study identifies life shifts and stress coping strategies in people with multiple sclerosis

**DOI:** 10.1038/s41598-022-10267-z

**Published:** 2022-04-20

**Authors:** Heidemarie Lex, Pollie Price, Lauren Clark

**Affiliations:** 1 Department of Psychiatry, University of Utah, 383 Colorow Drive, Salt Lake City, UT 84108 USA; 2 OTR/L, FAOTA, University of Utah College of Occupational and Recreational Therapies, Salt Lake City, USA; 3 FAAN, University of California at Los Angeles College of Nursing, Los Angeles, USA

**Keywords:** Health policy, Health care, Medical research, Neurology

## Abstract

Multiple sclerosis (MS) is an auto-immune disease in which the body’s immune system attacks the central nervous system. The demyelination of the nerve fibers can lead to physical, emotional, and cognitive impairments. We wanted to learn about challenges of living with the illness and how people deal with stress. 128 individuals with MS from Austria and the US participated in the qualitative interviews. We interviewed participants and coded their answers using inductive grounded theory. We asked three open-ended questions to inquire about life since being diagnosed with MS as well as about dealing with stress. Life shifts since diagnosis involved ‘experiencing limitations’ and could be categorized in ‘emotional changes’, ‘changes with work’, ‘changes in social interaction’, ‘physical changes’, ‘changes in the medical context’. For dealing with stress active (strategies and activities) and passive coping strategies (avoid/ignore) were employed. General stress reactions were expressed in areas of emotional, physical and /or lifestyle. We recommend developing interventions in three core areas for the MS population: (1) dealing with life changes and significant experiences with MS, (2) focusing on the areas where life shifts took place, (3) focusing on active coping with stress and discussing consequences of passive coping strategies.

## Introduction

Multiple sclerosis (MS) is a chronic auto-immune disease where the body’s immune system attacks its own central nervous system. Consequently, the demyelination of the nerve fibers can cause physical, emotional, and cognitive impairments. Living with MS is demanding, with both day-to-day and lifelong challenges. A continuous adjustment to life is required^1^.

Much qualitative research on this topic has been conducted by interviewing people with MS^2–6^. We decided to put the focus of our research on life shifts happening with MS and talked to people with MS about their experiences, life changes, and coping strategies to deal with stress.

It usually takes several years after diagnosis before persons with MS are able to interpret their illness experience in positive ways^1^. A high level of uncertainty about life and the future is experienced by persons with chronic illness, including MS. In addition, constantly changing body image following body and performance failures can lead to the loss of salient aspects of the self^6^. Women and men with MS expressed that they wanted to obtain mental health interventions immediately after the diagnosis, and that the inclusion of family members in both treatment and the whole experience of having MS was very beneficial for them^2,7^. With the physical consequences of the likely progression of MS, a continuous redefinition of identity is required^3^. Especially in the early stages of MS emotional and avoiding coping strategies are employed^5^. The necessity of information about emotional support for coping, the importance of social networks, and physical activity were shown as important for adjustment to secondary progressive multiple sclerosis (SPMS). Additionally it was found in this qualitative meta-analysis that adjustment to SPMS takes time^4^.

Stress experience is a significant aspect affecting health and happiness. There are factors that support health and influence the relationships between health, stress, and coping^8^. Health and illness are not static conditions; they are on a dynamic continuum^9^.There is evidence that stress affects health in the general population: 43% of individuals report adverse health effects from stress and 75–90% of all doctor visits are stress related. In addition, stress contributes to headaches, high blood pressure, diabetes, depression, anxiety, and many more health problems^10–12^. For persons with MS, emotional stress plays an important role and can lead to common symptoms such as depression, fatigue, and cognitive changes^13^. It was shown that stress plays an important role not only on the onset of MS, but also the frequency and intensity of stress and flair ups. Regarding depressive symptoms, it was shown that depression is part of the neurological symptoms of MS and that it is primed by peripheral inflammation while an acute neuroinflammation is happening^14^. It was shown by a meta-analysis by Zhang et al.^15^ that people with MS had significantly increased blood oxitative stress marker levels compared with healthy controls. A systematic review and meta-analysis showed an increased prevalence of depression and anxiety in MS^16^. For cognitive function in MS it was shown that it is affected by the level of disability and fatigue. No significant effect on cognition was shown by depression^17^. Regarding fatigue in MS it was shown in a systematic review that patient education programs, especially which were CBT (Cognitive Behavior Therapy) based, showed positive effects on reducing fatigue^18^. Oriented on literature about MS, we aimed to investigate life changes/shifts since being diagnosed with MS and how people with MS deal with general stress, and whether general stress was related to disease related stress.

## Method

We used the reporting guideline COREQ^19^ to report our qualitative study and results (Supplementary Information).

### Design

A qualitative, descriptive design using a series of guided semi-structured interviews was chosen to capture life changes due to MS and stress coping strategies among people with MS in Austria and the USA. The qualitative descriptive interview methods were informed by the research of Hellige and Sandelowski. Hellige^3^ conducted interviews with people with MS; Sandelowski is an expert on how to conduct qualitative research for quality of life and (chronic) illnesses^20^.

### Setting

In Austria, the sample was drawn from an inpatient neurorehabilitation clinic in Graz/Styria/Austria, which has a specialized physical-therapy facility for MS inpatients. In the US the interviews were taken in an outpatient medical center in Boston.

We selected Austria and the United States as study sites due to their high MS prevalence rates, which are higher than 100 per 100,000^21^ and their predominantly Christian, high-income countries using the World Bank classification system.

### Participants

Inclusion criteria for participation were age, a clinical diagnosis of MS, and the cognitive and expressive language ability to participate in a qualitative interview. For age, we limited participants to the age bracket of 18–57 to include only adults. We recruited 128 people with MS; more than two thirds of the participants had relapsing remitting MS and less than one third had progressive MS. In Austria we invited all people eligible to participate in the study which resulted in a sample of 64 participants. The interviews in the United States were conducted with outpatients at a multiple sclerosis clinic in Boston, MA. We wanted to have a comparable sample as in Austria and therefore included 64 participants from the US to our study.

In Table 1 an overview of the sample demographics can be seen.

**Table 1:** Sample demographics.

Demographics	Austria	United States
Participants	64	64
Women	39	39
Men	25	25
Mean age (18–57)	44.08 (*SD* = 8)	41.63 (*SD* = 8.64)
Mean EDSS^a^ (0: *no disability*, to 10: *death*)	4.54 (*SD* = 2.22)	2.88 (*SD* = 2.30)
Mean MS duration (years)	12.1 (*SD* = 7)	9.33 (*SD* = 7.4)
**Educational level, *n* (%)**		
No completed secondary education	5 (7.9%)	3 (4.7%)
Completed secondary education	48 (76.2%)	20 (31.3%)
Completed higher education/university	10 (15.9%)	41 (64.1)

^a^Expanded Disability Status Scale (EDSS).

### Data collection

In Austria and the US, the same female interviewer, the primary investigator, who is experienced in qualitative research, conducted all interviews, with those in Austria conducted in German and interviews in the United States conducted in English. The interview questions were carefully translated and back translated by independent parties into English for the US leg of interviews. In in-person interviews ranging from 20 to 40 min, participants with MS responded to open-ended semi-structured questions allowing for the possibility of multiple answers to each question and allowed interviewees to articulate their perceptions and experiences freely and spontaneously to more deeply understand experiences of change after a diagnosis of MS, experiences of living with MS, and ways of coping with stress. The questions were: ‘Which changes in your life have you perceived since you were diagnosed with MS?’, ‘Please describe your most significant experiences resulting from MS’ and “How do you cope with stress?’. We had no dropouts during the interviews. Interviews were audio taped and transcribed verbatim for analysis.

They were conducted using an interview guide that was checked within the research group and the questions are cited in the paper. No field notes were taken. No repeat interviews were carried out. No transcripts were returned or discussed with participants.

No one else was present in the room besides the participants and researcher during the interviews. The study participant and the interviewer had no relationship established prior to study commencement and participants knew about the purpose of the study and the personal interest of the researcher in MS. No one refused to participate.

### Data analysis

We applied convenience sampling. We started with data analysis after the interviews in Austria were conducted and can confirm that we reached data saturation because the same themes were reoccurring. We augmented the study with including a sample of 64 interviews from the US.

Including and analyzing our additional 64 US interviews allowed us to generalize our findings to a broader population.

We confirm that research including all methods were performed in accordance with the relevant guidelines and regulations. Trustworthiness of the data can be granted. Due to privacy protection of the participants, it was promised that their full-length interviews would not be published as these were open ended interviews and many things were shared.

The interviews were coded and analyzed by the female principal investigator, who is a sociologist and psychologist trained in qualitative research, who was in training for her PhD at the time of the interviews. A German speaking medical doctor, but English as native language and trained in conducting research, reviewed the transcripts and verified the translations in Boston prior to starting the interviews. The software MAXQDA^23^ was used to code the data.

We used Grounded Theory^24^ as the theoretical frame of our research; Thematic Analysis^26^ was our specific method. We used an inductive, descriptive approach called thematic analysis for data analysis^24–26^ whereby transcripts were coded in order to develop conceptual categories. After reading the transcripts in their entirety, we grouped statements dealing with similar topics in a category and refined them into an over-arching theme and subthemes.

We present participant quotes with the interview number, participant sex, and participant nation in parentheses; for example, ‘I34, M, U.S.’ identifies a quote from Interview 34, in which the participant was male in the United States. Categories are supported by participant quotes. Transcripts and results were not shared and discussed with participants. No feedback was given. Regarding a description of diverse cases opposing interview quotes are presented in the findings. In the findings, we also present a table with a coding/category tree.

### Ethical approval

In Austria and the US, the Ethics Commission of the associated medical center in Graz/Austria respectively the Institutional Review board (IRB) of the Beth Israel Medical Center, a Teaching Hospital of the Harvard Medical School in Boston/US, approved the study and interviews and consent forms. We provided participants with both written information and an oral explanation of the study and qualitative interviews and obtained their signed consent^22^. We determined their cognitive and language ability by having them sign the consent. See Table 1 for the demographics of the participants. This study was performed in line with the principles of the Declaration of Helsinki. Approval was granted by the Ethics Committee of the Medical University Graz/Austria in 2010 and the IRB in Boston/US in 2013.

### Consent to participate

All interviewees gave written consent to participate in the study and the interviews, the study was IRB approved in Austria and the US.

## Findings

Findings revealed two overarching themes (l) life shifts through MS and (2) dealing with stress. Table 2 gives an overview of the detailed qualitative results, which are described in the following:

**Table 2:** Qualitative interview results—coding/category tree.

	Subcategories/codes	Examples
Life shifts: overall theme: experiencing Limitations	Emotional changes	Crying, disappointments
	Changes in work	Hours employed
	Changes in social interaction and lifestyle	Relationships to friends, family and activities
	Physical changes	Strength, endurance
	Medical context	Medical experiences
8 General stress coping strategies	Active coping; (1) strategies and (2) activities	Strategies: planning how to react, stepping back, Activities: meditate, go on a walk or run, meeting friends
	Passive coping; (3) avoid; (4) ignore; (5) no strategies	Don’t deal with stress
	(6) emotional; (7) physical 8) lifestyle reactions	Emotional reaction: crying, nervousness
		Physical reaction: weakness
		Lifestyle reaction: alcohol, cannabis, changes in lifestyle (e.g. nutrition, sleep)

### Life shifts through MS

We were interested in life with MS, specifically what changed most notably, and what experiences were significant as a result of MS. The theme, life shifts through MS, resulted as the first two questions, which had similar emergent themes, were analyzed together.

The overarching theme under which the other subthemes fall is *experiencing limitations*.

Within *experiencing limitations,* participants described changes in *emotions, work, social interactions and lifestyle, and physical status*. Below are participant quotes for the identified theme and subthemes:

*Experiencing limitations* described constraints through MS. It included statements such as ‘I feel like I've slowed down. The fatigue has made me not be able to do as much as I would like to do. I'm just much more aware of taking care of myself and what can I do to myself and doing what I can to make sure that I don't have stress, taking care of myself.’ (I32, F, US).

Someone else stated ‘That I am not that mobile anymore. That I can’t do certain things anymore (I60, F, AUT). Another one expressed ‘Lack of being able to do things I wanna do. I can’t see very well, I can’t drive any more, I can’t read things like I used to—has to be read to me or I have the letters enlarged, so that I can read them. Cook: I can’t follow a recipe, I just through things together. (I50, F, US). One person stated ‘I did not have negative experience with MS. Everyone who knows me doesn’t believe it (I49, F, AT).

*Emotional changes*: This sub-theme described what changed emotionally for participants since they were diagnosed MS. This included statements such as ‘…then I get nervous, I panic (I08, M, AT) and ‘I guess, whatever is happening during the flare up symptoms. And that brings to the surface uncertainty about the disease. Most notably, I couldn't write and when I wrote, it was like a child. And it was very uncertain at that time. I didn't know, is it gonna be better or not get better. It’s always around flare ups. Maybe realizing once again, how uncertain everything is. It could change every moment.’ (I23, M, US). Another one stated ‘The whole life changes with MS, now I am also depressed from time to time’ (I38, F, AT).

*Changes in work*: This subtheme summarized what people expressed about their work adaptions. Answers in this subtheme included statements such as ‘The job, that's the biggest one. The fact that I had to stop working. That's the biggest one. The fact that I couldn't do the job any more is still very hard for me.’ (I65, F, US). Another participant stated: ‘My lack of being able to work as hard as I used to. Sometimes I just get frustrated because I can't go as fast or do as much as I used to. I don't have the ability to do what I used to do.’ (I59, M, US). Another one stated'.

Again, I had to choose a different career path (I10, F, US).

*Changes in social interaction and lifestyle:* This subtheme described what social changes in their interactions and their whole life with MS were reported. One participant stated: ‘I'm definitely less social than I used to be. It feels unpredictable to make plans with other people because often I feel very tired. And it just became very frustrating that you have to cancel plans, so I avoid making plans.’ (I43, F, US). Another stated: ‘You are not being accepted anymore. My circle of friends has changed. When you can't participate anymore, they leave you. Healthy people can't accept your situation even if they try. Other people with MS can do that.’ (I52, M, AT). Another person stated ‘my social environment does not react at all because they don’t know what that is. But many react with Oh, Oh, …that scares me (I01, M, AT). Another one stated ‘That I had good friends which are not really good friends. But I also won new friends; (I25, F, AT). Another one stated ‘my inner family circle (children, husband) were very scared at the beginning, but talking about it helped. My husband got more supportive (I41, F, AT).

Another one said ‘my amount of physical employment changes. I have more leisure time, we changed our house building plans, I changed my nutrition and I think about physical activity (I54, F, AT). Another person said ‘Changed my whole lifestyle. Everything fun I can’t do anymore, especially Sports. Family became more important, because they try to support me. Quality of Life got much worse.’ (I64, M, AT).

*Physical changes*: The sub-theme *physical changes* was defined by physical transformations expressed due to MS. It included statements such as ‘I am not as active physically. I am still as active mentally, maybe even more, because I'm determined.’ (I10, F, US). Another participant stated: ‘Physically it made me weaker but mentally it made me stronger. I found my voice, it's weird how it happened, but I really did’ (I14, M, US). Another statement was ‘Do more exercises, probably more knowledge of the disease. And do more research. Read of new medication, studies.’ (I02,M, US). Another one stated ‘Not being able to walk. I keep a cane at my leg sometimes to be able to walk. Sometimes I have no strength in my leg. (I02.M,US).

Another person stated ‘I have to be careful when I walk downstairs, I get fatigue, I have to stop and sit, rest’ (I38, M, US).

*Medical context* includes experiences made with medical personnel or with medication. One statement of this category is ‘(I had) negative experience with nurses in the hospital. They treat you as if you were only a number …and the way they treat people … very shocking (I12, F, AT). Another one stated ‘I was on (medication) Copaxone, then I was switched over to medication Tecfidera. I am really happy not taking Copaxone anymore. The shots made me very emotional. And having bruises. People could actually see about that, made me very sad.’ (I15, F, US). Another one stated ‘I am just more aware towards the end of the week. As I inject Thursday night, you know my Fridays keeping them a little more low, sort of low key, in sort of managing the side effects of the medication, which I continue to have. Headaches and flu like symptoms are manageable with Advil but I also try to keep my activity level at a more manageable level on towards the end of the week, following the injections.’ (I34, F, US). Another one stated ‘It was the first medication, that didn’t agree with me. I felt how it is to not have your ability to move, like it does affect your legs in weird ways and that was a little scary. But changing the medication, that helped.’ (I39. F, US).

### Managing general stress when having MS

We identified eight different categories for coping with general stress. They were further categorized as active and passive coping. Active coping included both, strategies and activities, to cope with general stress.

#### Active coping with stress

Active Coping included active stress coping strategies and active coping activities. Active Coping was defined as self-initiated effort to reduce triggering circumstances or exposure to environments that were difficult. *Active stress coping strategies* were defined as all activities that help dealing with stress actively, by using strategies that were helpful in the past and will therefore be used again*:* one participant explained the importance of planning: ‘Making a plan, having things written down so that I know what I'm doing. I sort of take a step back and write down what's happening and how to deal with it.’ (I40, F, US). Another participant stated: ‘The best way that I can. Honestly, I pretty much stay quiet, stay calm and do my own things. If I wanna go for a walk or take a drive somewhere, I do that. I just try to separate myself from what the problems are. Try not to get overexcited about things, because stuff can always be fixed.’ (I27, M, US). Another one expressed ‘Very well. I guess I know what’s gonna happen and I realized that fact and I accept it (I07, M, US). Another one stated ‘I try to just think about it, let it go, try to think there is something better than what I’m stressing out about. Try to figure out how to correct what’s stressing me out. It’s a hard one (the question). (I59, M, US).

*Active stress coping activities* is a subcategory of active stress coping strategies, and they overlap in many cases. They were defined as activities that someone performs to reduce stress. Answers in this category include statements such as ‘I write a lot, I create on so many levels, I paint, I sculpt, I have friends to talk to.’ (I14, M, US), or ‘I try to schedule times with friends for a walk or dinner. I'm in a group of friends reading books and we meet every 2 months.’ (I25, F, US). Another one stated ‘So I just got another dog, so I have two little dogs and they help, unconditional love. I go to the beach. I love the beach, just the sound of the ocean and I watch TV. (I61, F, US).

#### Passive coping with stress

Passive Coping was defined as having no positive stress coping strategy or activity in place at all.

We identified several passive coping themes; these included: try to avoid stress, no strategies, ignoring stress, emotional stress reaction, physical stress reaction, and lifestyle reactions.

*Try to avoid stress*: This category was defined by using no strategies to deal with stress, other than trying to avoid triggers that lead to stress. One participant stated: `I avoid stress as much as possible and try to stop it from happening' (I10, F, AT). Another participant said: ‘I just bottle it in and it comes out to a later time. I was always like that.’ (I05, M, US). Another one said ‘I try to avoid stress’ (I38, F, AT).

*No strategies*: This category was defined by having a lack of ideas of how to deal with stress. One participant stated `I always try to say it’s gonna be ok, it’s gonna be ok, but in the back of my mind I'm wondering if it's really going to be ok. I tend to overthink things.' (I13, F, US). Another one said ’(P speaks rapidly and loud) I don’t, I don’t, I really have a hard time dealing with that, I fall apart. (I01,F, US). Another one expressed ‘but I have no strategies at the moment, to deal with stress’. (I28, F, AT).

*Ignoring stress*: Ignoring stress was defined to consciously try to ignore the stress while *try to avoid stress* describes that no strategies were used to deal with stress other than to avoid dealing with it. One participant stated: `I try to relax and ignore it. Whatever stresses me out I try to ignore it.' (I42, M, US). Another person stated ‘I try to ignore stress, although for work it does not always work, as everything has to be completed on time’ (I09, M, AT).

#### Stress reactions

*Emotional stress reactions* were defined as emotional reaction as a result of being stressed out. It included for example statements such as ‘Stress is like poison for me. I get nervous, insecure.’ (I27, M, AT). Another person expressed ‘then I fall into my old pattern again, telling myself that I have to do everything on my own, that I can’t accept help’ (I24, F, AT). Another person said ‘I put lots of pressure on myself. It is less stress from the outside, more my own expectations from myself’ (I07, M, AT).

*Physical stress reactions* were defined as how someone’s body reacts to stressors. This category included statements such as ‘I don't like stress, it makes me not feel very good, I get symptoms: numbness, tingling, I can't walk right.' (I52, F, US). Another one stated ‘I feel physically immediately worse’ (I56, F, AT).

*Lifestyle reactions* describe how people reacted to stress in their lifestyle, e.g. drinking alcohol, smoking or how they changed their lifestyle (e.g.nutrition, sleep). It included statements such as ‘Ahm, ahm, marijuana, I do tend to drink a little bit, I spend money, I eat.’ (I12, M, US). Another person stated ‘I don’t take everything that serious. I don’t have to be everywhere anymore. When I am on vacation, I don’t have to visit every sight around there. We have been everywhere before, now we have children and that is important’ (I09, M, AT). Another one said ‘I breathe, count to 10 and maybe smoke cigarettes’ (I39, F, US).

## Discussion

To the best of our knowledge, this is the first qualitative study assessing life experiences with MS in such a large sample size using qualitative semi-structured open-ended questions.

The aim of our study was to dive deeper into the meaning of having to live with MS, and what changes and experiences formed the life of people with MS. Hellige^27^ talked to 18 people with progressive MS in 2002 and found that life changes over time happen to people with MS, which are demanding and challenging (for the present and the future). We interviewed 128 people with MS and found explicit changes in life (so called life shifts), which confirmed Hellige findings, and could specify that these took place in six areas, namely ‘experiencing limitations’, ‘emotional changes’, ‘changes with work’, ‘changes in social interaction’, ‘physical changes’, ‘changes in the medical context’ (see results section for more detail). We decided to discuss the three areas ‘changes in ‘emotions’, ‘work’, ‘medical context’ in more detail, as we could add additional findings to current research on these topics.

In addition, we could show in our results ways how people with MS cope with their lives when dealing with general stress and we will discuss possible future directions based on these results.

### Life shifts through MS

#### Emotional

Literature, especially by Kalb^28^, dealt with the psychological impact of MS, when cognitive changes take place in MS or when relapses happen. The author described that acute relapse of MS are experienced as crises including grief, anxiety, anger, and guilt.

In recent literature^15,16^ an increased prevalence of depression and anxiety in MS could be found. Our results showed that emotional reactions and anxiety were expressed due to a sudden physical change during for example experiences of MS symptoms. As was shown by Dehghani et al.^29^**,** the maintenance of emotional balance, acceptance of the disease and self-regulation are favorable factors for coping with MS. Our results as well show that emotional reactions related to general stress were expressed frequently. A new result we could add to literature is that emotional reactions to general stress seem to be a way to cope in persons with MS.

#### Work

The authors Beier et al.^30^ showed that the factor of employment status causes stress in persons with MS. Dortsyn et al.^31^ found in their meta-analysis that employed people with MS reported significantly greater quality of life and mood, cited fewer work and MS difficulties and were more likely to adopt problem-focused coping strategies. In their meta-analysis Gerhard et al.^32^ recommend that work interventions for people with MS should help be aimed at enabling them to remain in workforce and accommodate and facilitate functional independence.

In our study, changes in work were reported frequently as a (negative) life shift due to MS. The fear of losing work respectively structure was expressed often. We want to add to literature that people with MS suffered due to the loss of work and structure. They were willing to work (but not the full amount of time was possible). Diminished self-worth and negative coping were expressed frequently in this context.

#### Changes in the medical context

Wagner et al.^33^ showed that the growing number of people with a major chronic illness face many obstacles in coping with their condition, also within the health care system. Medical care often does not meet their needs for effective clinical management, psychological support, and information. Particularly poor communication with healthcare professionals and medication was reported frequently in our interviews with people with MS.

### Stress

Our results add new insights to the understanding general stress coping strategies of people with MS. Passive coping strategies were expressed frequently. Emotional, physical and lifestyle reactions to general stress were identified as ways to cope with stress for persons with MS.

### Implications for intervention

Our research could be used to develop interventions where the areas *emotional, work, social interaction and lifestyle, physical aspects of life and the medical context* could be focused on*.* Sanchez et al.^34^ identified needs and demands of people with MS as well. They could show the importance of empowering people with MS.

We recommend that people with MS as well as their professional and personal support persons should be included in developing interventions as well as trainings focusing on these interventions^2,7^. Rintell et al. could show the importance of including family in intervention planning for MS and the early wish of Persons with MS to get mental health support. In our interviews more support from family, friends and medical personnel was expressed frequently.

Our findings should be used for psychoeducational trainings about stress and coping with life shifts, accepting and managing limitations^35,36^. Support should also be focused on planning goals, identifying meaning in life, as well as accentuating cultural and personal strengths.

Lastly, we recommend addressing the meaning of passive coping strategies and their importance in the context of emotion regulation. Emotional stress reactions should be addressed in MS. Learning about emotion regulation and its consequences should be the goal when planning effective and healthy life style, also to improve quality of life, as well stated by Phillips et al.^37^.

### Study limitations

A potential source of bias is the recruitment strategy (inpatients in Austria vs. outpatients in the US), which differed for practical reasons. Inpatient rehabilitation centers are not offered in a similar amount in the US as in Austria due to differences in the social security system.

In addition, we sampled more participants than we would have needed to reach data saturation. However, out of respect towards invited MS patients, we wanted to give all of them interested in sharing their thoughts the opportunity to do so.

Because we collected data in only two settings, we cannot claim that our findings generalize to the whole MS population. Although, the large sample size does add to the rigor of the findings. A limitation of the data analysis is that there was only one person coding the interviews and analyzing the data.

We did not directly ask participants about their quality of life and the relationship between quality of life and other concepts such as coping with stress due to limitations of time (how long study participants would be ok to be interviewed. We did not want to exceed 50 min). It will be important for these two topics to be addressed in future research.

We also did not specifically address early onset MS and are aware that this type of MS carries its own specific challenges and burdens. Future research should focus on how challenges with the onset of MS are perceived and how experiences evolve over the lifespan, taking into consideration factors such as duration of disease, level of impairment, marital status, having children, job satisfaction, and communication styles, among others.

We did not set a focus on healthcare providers and their needs. This should be the goal for future research in this area—focusing on challenges and needs of healthcare workers.

## Conclusion

Based on our findings, we recommend developing psychological and social interventions in three core areas for the MS population: (1) dealing with life changes and significant experiences with MS, (2) focusing on three out of many areas detected where life shifts took place: emotional, work and aspects in the medical context of persons with MS. (3) focusing on active coping with stress as goal for people with MS, within their social and medical interactions, and making passive strategies/coping and emotion regulation a subject of discussion.

## Supplementary Information

Supplementary Information.

## Data Availability

Interview data was collected for the PIs dissertation. Contact the PI for more information.
